# The Association Between Periodontitis, Gingivitis, Tooth Loss and Stroke: An Umbrella Study with Meta-Analysis

**DOI:** 10.3390/brainsci15010010

**Published:** 2024-12-26

**Authors:** Jad El Masri, Ahmad Al Malak, Diala El Masri, Maya Ghazi, Silva Al Boussi, Yasmina El Masri, Mohammad Hassoun, Maryam Tlayss, Pascale Salameh, Hassan Hosseini

**Affiliations:** 1 INSERM U955-E01, Institut Mondor de Recherche Biomédicale (IMRB), Université Paris-Est Créteil, 94010 Créteil, France; consultation.hosseini@gmail.com; 2 École Doctorale Sciences de la Vie et de la Santé, Université Paris-Est Créteil, 94010 Créteil, France; 3 Faculty of Medical Sciences, Lebanese University, Beirut 1533, Lebanon; mayanghazi99@gmail.com; 4 Neuroscience Research Center (NRC), Faculty of Medical Sciences, Lebanese University, Beirut 1533, Lebanon; diala.s.masri@std.balamand.edu.lb; 5 INSPECT-LB (Institut National de Sant e Publique, d’Épidemiologie Clinique et de Toxicologie-Liban), Beirut 1103, Lebanon; pascalesalameh1@hotmail.com; 6 Faculty of Dental Sciences, Lebanese University, Beirut 1153, Lebanon; ahmadmalak56@gmail.com (A.A.M.); yasminamasri03@gmail.com (Y.E.M.); mohamadmonahassoun@gmail.com (M.H.); 7 Faculty of Medical Sciences, University of Balamand, Koura 1100, Lebanon; 8 Department of Internal Medicine, American University of Beirut Medical Center, Beirut 1107, Lebanon; bosisilva@gmail.com; 9 Faculty of Arts and Sciences, University of Balamand, Koura 1100, Lebanon; maryamtlayss.tlayss@std.balamand.edu.lb; 10 Faculty of Pharmacy, Lebanese University, Beirut 1153, Lebanon; 11 School of Medicine, Lebanese American University, Byblos 1102, Lebanon; 12 Department of Primary Care and Population Health, University of Nicosia Medical School, Nicosia 2417, Cyprus; 13 Department of Neurology, Henri Mondor Hospital, AP-HP, 94010 Créteil, France

**Keywords:** cerebrovascular diseases, stroke, periodontal diseases, periodontitis, tooth loss

## Abstract

Background: Cerebrovascular diseases (CVAs) have several risk factors that are categorized as modifiable and nonmodifiable. Periodontal diseases (PD) have a modifiable role in causing CVA, where several studies suggested direct or indirect correlations with systemic diseases. This study aims to summarize, evaluate and analyze all the evidence available in literature, to reach a better understanding of the relation between periodontitis, gingivitis, tooth loss and CVA. Methods: PubMed, Cochrane, Scopus and Web of Science databases were searched for all meta-analyses assessing the effect of PD on CVA in accordance with Joanna Briggs Institute guidance for umbrella reviews in March 2024. Assessment of Multiple Systematic Reviews (AMSTAR) was used for quality assessment. Pooled analysis was performed to assess the effect of periodontitis, gingivitis and tooth loss on CVA, depending on the availability of data using Review Manager Version 5.2.11. Results: Seven of the identified meta-analyses were of high quality, and they were distributed in different countries. Periodontitis was generally associated with a significant increase in CVA risk (OR = 2.32, 95% CI: 1.70, 3.17, *p* < 0.00001 and RR = 1.22, 95% CI: 1.15–1.29, *p* < 0.00001), same as tooth loss, but to a lower degree (0.78). However, the effect of gingivitis was insignificant in terms of CVA (RR = 1.32, 95% CI: 1.12–1.56, *p* = 0.0008). Conclusions: This study confirms the effect of periodontitis and tooth loss on CVA, disregarding any significant role for gingivitis. The relation reached favors the suggested role of some inflammatory changes in the pathogenic pathway leading to atherosclerotic changes.

## 1. Introduction

Cerebrovascular accidents are the leading cause of adult disability and the second leading cause of death, with a high prevalence worldwide [1]. Risk factors for stroke are numerous and categorized as modifiable and nonmodifiable. Hypertension, smoking, diet, and physical inactivity are the main reported modifiable risk factors, and atherosclerosis is one of the most important causes of stroke [2,3].

Another modifiable risk factor for CVA is the presence of periodontal diseases. Several studies suggested a direct or indirect correlation between periodontal diseases and systemic diseases, such as diabetes and cardiovascular and cerebrovascular diseases, which are also considered risk factors for CVA [4,5].

Periodontal diseases (PD) are inflammatory diseases expressed due to genetic, immunological components, and local or psycho-social factors [6]. There are various forms of PD, the primary of which is gingivitis resulting in inflammation and bleeding of the gingiva. If left untreated, this can lead to periodontitis characterized by gingival-attached connective tissue destruction and alveolar bone loss [7]. PD has a high prevalence worldwide, where more than a billion (19%) of the world population have severe PD [8].

Regarding stroke, an indirect relation with periodontitis was suggested mainly due to the inflammatory process. Inflammatory sites, such as the gingiva, periodontal ligaments or the bone in the oral cavity, are highly rich in blood supply via lingual, maxillary and facial arteries, emerging from the external carotid branches [9]. Atherosclerosis is the main mechanism through which periodontitis can cause CVA. This can occur through several routes, the first of which is by bacterial translocation into the systemic circulation. This leads to bacteremia and eventually promotes atherogenesis [10]. The second mechanism is mediated by inflammatory cytokines produced during periodontitis, including tumor necrosis factor (TNF), interleukin-1β (IL-1β), and IL-6. These mediators escape into the circulation and lead to an acute-phase response in the liver where CRP, fibrinogen and serum amyloid A become markedly elevated and promote atherogenesis [11,12]. Another mechanism participating in CVA is the effect of *Porphyromonas gingivalis* on the gut microbiota, which indirectly causes systemic inflammation, and therefore promotes atherogenesis in a similar mechanism to what is mentioned above [13]. In addition, periodontal inflammation could activate platelets and lead to increased coagulation factors, predisposing to stroke [14].

Several systematic reviews with meta-analysis suggested a correlation between PD and stroke [15], while others showed no significant link [16]. Considering the variation in results and the differences found by previous reviews and taking into account the high prevalence of PDs and tooth loss and its possible relation to CVA, this study aims to summarize, evaluate and analyze all the evidence available, leading to a better understanding of the relation between periodontitis, gingivitis, tooth loss and stroke.

## 2. Methods

An umbrella review with meta-analysis was conducted in accordance with PRISMA guidelines and the Joanna Briggs Institute guidance to examine the effect of periodontitis, gingivitis and tooth loss on causing CVA [17,18,19].

### 2.1. Search Strategy and Study Selection

A literature search was conducted in March 2024 by 2 reviewers (AM and JE) using the following electronic databases: PubMed, Web of Science, Scopus and Cochrane. Using Boolean operators, the following terms were used in the search: (Periodontal diseases OR Periodontitis OR Gingivitis OR Tooth loss OR Gum diseases) AND (Stroke OR Cerebrovascular accident OR Brain injury). Additionally, authors checked references and citations using snowballing techniques to reach additional papers. The title and abstract of potential eligible studies were screened by the two reviewers (AM and JE) independently, then the complete text of potential eligible studies was reviewed by the same investigators and disagreements were resolved by thorough discussion with a third reviewer (MG). All excluded studies with potential eligibility were either excluded due to the absence of a meta-analysis or they did not conduct a meta-analysis on the relation between stroke and periodontitis and teeth loss

Systematic reviews with meta-analysis were included in this umbrella review. After removing all duplications, original papers retrieved from all included systematic reviews were used in the meta-analysis.

### 2.2. Eligibility Criteria

Only systematic reviews with meta-analysis or meta-analysis reviews related to the association between periodontitis, gingivitis, tooth loss and CVA were included without any language or publication year restrictions. Exclusion criteria included all animal or in vitro studies in addition to papers not related to the association between stroke and periodontitis and teeth loss. Pico definitions: 1—Population: Patients of any age, gender, case background, country and ethnicity; 2—Exposure: periodontitis, gingivitis or tooth loss; 3—Comparison: controls having no periodontitis, gingivitis or tooth loss; 4—Outcome: all forms of CVA.

### 2.3. Data Extraction

Using an Excel spreadsheet, the two investigators mentioned above retrieved independently from the meta-analysis, the following information: (1) number of studies involved, (2) number of patients, (3) study main aim, (4) year, (5) country, (6) source of funding, (7) heterogeneity of small studies and (8) conflict of interest. The significance, conclusions and quantitative values reached by each paper were also summarized. 

### 2.4. Quality Assessment

The quality of each systematic review with meta-analysis included in this umbrella review was evaluated using the AMSTAR (Assessment of Multiple Systematic Reviews) tool, which is an 11-item instrument used for quality assessment of meta-analyses, rating them as low, moderate or high-quality reviews [18].

### 2.5. Data Analysis

After retrieving all original papers included in all the systematic reviews, pooled analysis was performed to compare the incidence of CVA between study groups, depending on the availability of data. A random-effects model was selected to account for statistical heterogeneity across the included studies using Review Manager Version 5.2.11 (The Nordic Cochrane Centre, The Cochrane Collaboration, Copenhagen, Denmark). The I2 (variability) statistic is the percentage of total variation across studies due to heterogeneity. *p* values less than 0.05 were considered statistically significant (*p* < 0.05). Odds ratios (ORs), hazard ratios (HRs) and relative risks (RRs) were used in different studies to quantify the risk of CVA. Forest plots were generated and presented for the following exposures: periodontitis, gingivitis and tooth loss.

## 3. Results

### 3.1. Research Yield

**Figure 1:**
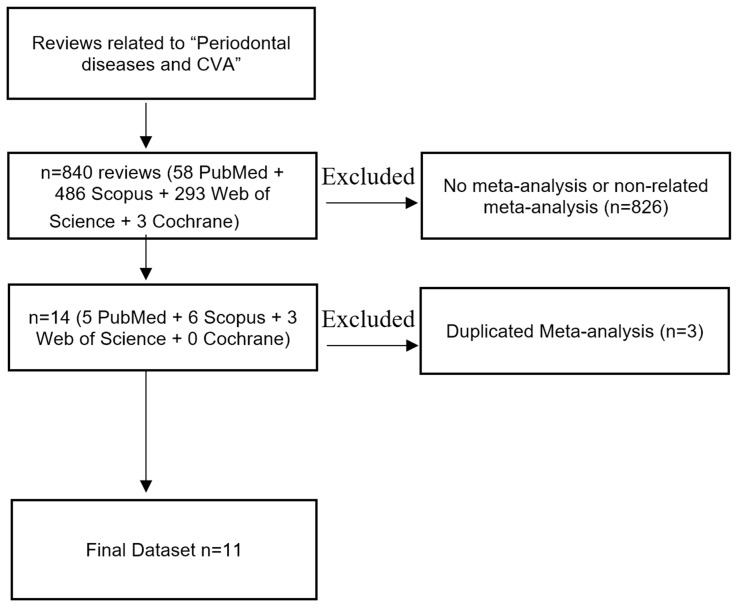
Prisma flow diagram of the selection process.

A total of 840 studies were found using an electronic search of the databases mentioned above. After the deletion of 829 studies with no meta-analysis and studies with meta-analysis with no correlation regarding stroke and periodontitis and teeth loss, 14 papers were eligible for the included criteria. After the elimination of duplicates, this umbrella included 11 meta-analyses evaluating stroke in periodontitis, gingivitis and tooth loss (Figure 1).

Table 1 summarizes the characteristics of each meta-analysis included in this Umbrella review. The 11 meta-analyses included were published between the years 2003 and 2023; two studies took place in China, two in the United States and the remaining studies were in Jordan, France, UK, Greece, India, Brazil and Spain. Regarding the number of studies, it ranges from 2 studies, as in the study by Janket et al., 2003 [20] to 19 studies, as in the study by Larvin et al., 2021 [21], Leng et al., 2023 [22], and Dewan et al., 2023 [23]. The majority of the studies had no funding and no conflicts of interest. Seven reviews were of high quality, four of moderate quality, and none were of low quality.

Table 2 clarifies the main aim of each meta-analysis and its conclusion, where most of the studies assessed the relation between periodontal diseases and the risk of stroke, showing a positive correlation. Some studies also assessed the effect of PD on cardiovascular diseases besides CVA. As for their conclusions, the majority highlighted a role for PD in causing CVA, yet some found no effect.

Table 3 provides the quantitative results found by each meta-analysis regarding periodontitis, gingivitis and tooth loss and the risk of stroke, specifying the *p* value and heterogeneity of each review. The majority of studies found a significant correlation between PD and CVA, while four studies showed no significant correlation: Mustapha et al. [16], Leng et al. [22], Lafon et al. [26] and Dewan et al. [23].

### 3.2. Periodontitis

#### 3.2.1. Case-Control Studies

**Figure 2:**
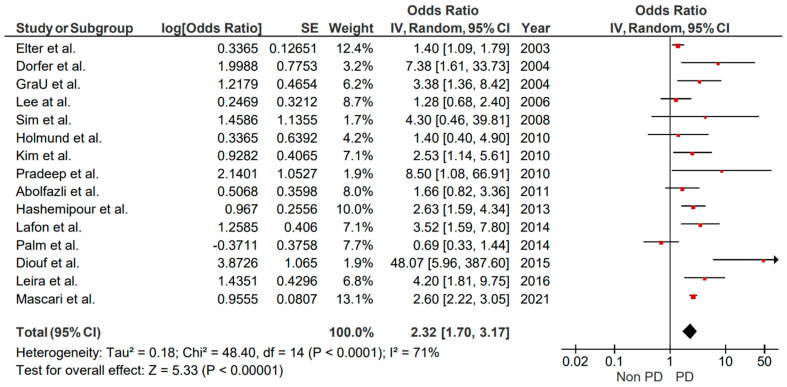
Forest plot illustrating results from random effect meta-analysis for the incident risk of CVA in patients with periodontitis (case-control studies).

**Figure 3:**
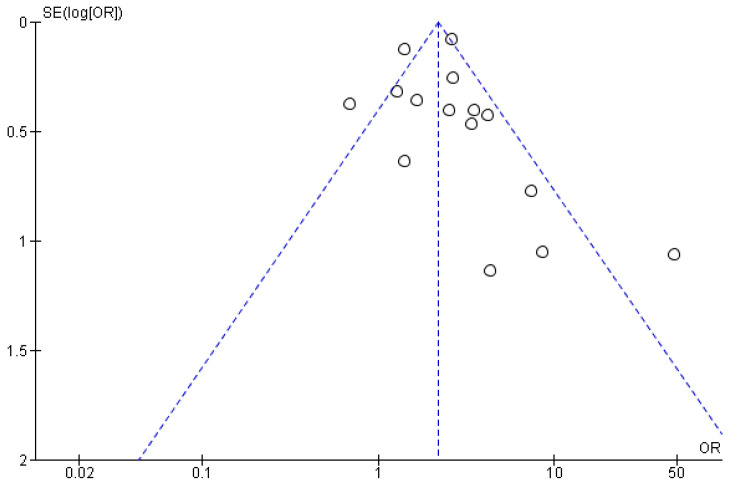
Funnel plot for case-control studies targeting the incident risk of CVA in patients with periodontitis.

Original papers assessing the relationship between periodontitis and stroke were 15. The overall heterogeneity was high (I^2^ = 71%). The pooled results showed a significant association between periodontitis and stroke, where individuals suffering from periodontitis had around two times higher chance of developing stroke than those without periodontitis (OR = 2.32, 95% CI: 1.70, 3.17, *p* < 0.00001) (Figure 2*).* No asymmetry was detected throughout the visual examination of the funnel plot targeting the relation between stroke and periodontitis in case-control studies, suggesting the possible absence of small study bias (Figure 3).

#### 3.2.2. Retrospective Cohort Studies

**Figure 4:**
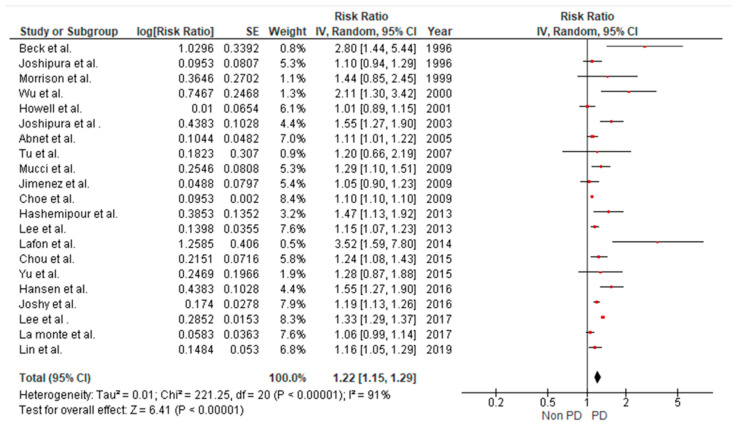
Forest plot illustrating results from random effect meta-analysis for the incident risk of CVA in patients with periodontitis (retrospective cohort studies).

**Figure 5:**
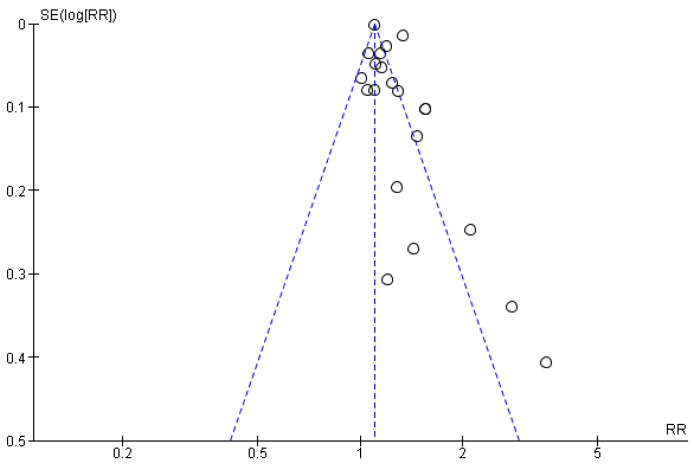
Funnel plot for retrospective cohort studies targeting the incident risk of CVA in patients with periodontitis.

The pooled results of the 22 studies included showed substantial heterogeneity with I^2^ = 91%. Results were significant and showed an association between periodontitis and stroke (RR = 1.22, 95% CI: 1.15–1.29, *p* < 0.00001) (Figure 4). Asymmetry was detected throughout the visual examination of the forest plot targeting the relation between stroke and periodontitis in retrospective cohort studies, suggesting the possible presence of small study bias (Figure 5).

### 3.3. Gingivitis

**Figure 6:**
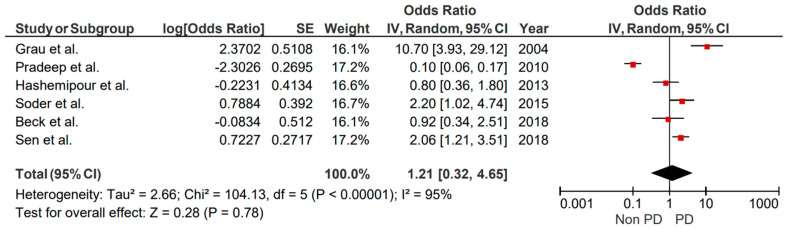
Forest plot illustrating results from random effect meta-analysis for the incident risk of CVA in patients with gingivitis (case-control studies).

The six studies eligible for the assessment of gingivitis to the risk of stroke were of high heterogeneity (I^2^ = 95%), and data pooled to form the forest plot revealed a remarkable difference between the odds ratio (OR) of the studies included. According to our findings, there is no significant association between gingivitis and the risk of stroke (*p* = 0.78) (Figure 6). Small studies biases reporting could not be conducted, as <10 studies were included.

### 3.4. Tooth Loss

#### Retrospective Cohort Studies

**Figure 7:**
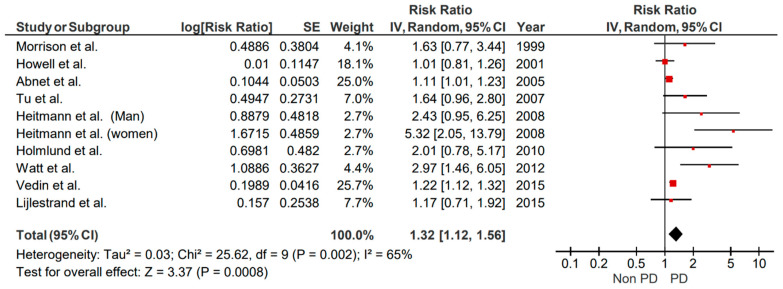
Forest plot illustrating results from random effect meta-analysis for the incident risk of CVA in tooth loss (retrospective cohort studies).

Ten original studies assessed the presence of a relationship between tooth loss and stroke. The overall heterogeneity was moderate (I^2^ = 65%). Moreover, the combined results of each study revealed that tooth loss and the risk of stroke are correlated (RR = 1.32, 95% CI: 1.12–1.56, *p* = 0.0008) (Figure 7). Small studies biases reporting could not be conducted, as <10 studies were included

## 4. Discussion

This study provides a wide review summarizing all studies attempting to relate periodontal diseases to CVA to date. Therefore, we offered an all-inclusive umbrella review and a meta-analysis of studies with compatible methodology in evaluating outcome variables. Notably, seven of the identified meta-analyses were of high quality, and they were distributed in different countries. According to our results, PD was generally associated with a significant increase in CVA risk, as well as tooth loss, yet to a lower degree. However, the effect of gingivitis was insignificant in terms of CVA incidence.

Regarding gingivitis, which is a milder form of inflammation compared to periodontitis, this study found no significant relation to CVA risk, despite some of the retrieved studies claiming it is protective, with others claiming that it is harmful. This could be due to the low levels of inflammation, causing less atherosclerotic changes [7]. The variations recorded in different studies could be attributed to different populations included, as individuals predisposed to atherosclerosis and its risk factors are logically more prone to being affected by gingivitis, even with its milder levels of inflammation compared to periodontitis [29].

As for tooth loss and its positive correlation with CVA, the main mechanism is believed to be through the increased risk for tooth loss following periodontitis or PD [30]. One cause for tooth loss can be advanced periodontal disease, causing inflammation-associated atherogenesis. This can account for the increased risk of CVA [31]. Moreover, tooth loss can be associated with nutritional changes, mainly to soft, easier-to-chew foods [32]. On average, patients also have a decreased intake of vegetables and fruits, which are proven to be protective against ischemic events [33]. The altered diet can eventually lead to increased triglyceride, cholesterol and LDL levels, which in turn predispose to cardiovascular disease [34]. Tooth loss, when resulting from dental caries, is usually due to high carbohydrate intake, and this further explains the increased risk for CVA associated with tooth loss, as carbohydrate intake was shown to increase the risk for stroke [35]. Lastly, tooth loss disrupts the integrity of the periodontal tissue allowing the oral microbiota to reach the systemic circulation, therefore leading to inflammation [36].

Finally, this study has several limitations. For instance, this study is susceptible to publication bias, where studies with positive outcomes are more likely to be published, leading to a skew in the results. Furthermore, findings include high heterogeneity levels, possibly due to the lack of separation between ischemic strokes, hemorrhagic strokes and transient ischemic attacks. Also, despite having some common etiologies and risk factors, the lack of classification of CVA cases could disrupt the understanding of pathogenesis. However, most original papers and systematic reviews combined them as one disease. Another limitation worth mentioning is the lack of assessment of confounding variables, mainly cardiovascular risks, as data were not available in original studies.

## 5. Conclusions

The findings of this umbrella review with meta-analysis confirm the effect of periodontitis and tooth loss on CVA, disregarding any significant role for gingivitis. The relation reached favors the suggested role of some inflammatory changes in the pathogenic pathway leading to atherosclerotic changes, where the lower levels of inflammation in gingivitis compared to periodontitis limits its atherosclerotic effects, and the inflammatory process following tooth loss leads to a comparable effect to CVA incidence in the presence of periodontitis. Further research should focus on understanding the specific mechanisms, with a special focus on separating CVA cases into specific categories such as ischemic, hemorrhagic and transient. Also, future research should standardize reporting by adjusting confounding factors, mainly cardiovascular risk factors, through conducting some prospective studies with robust statistical modeling.

## Figures and Tables

**Table 1:** Overall characteristics of meta-analyses included in the umbrella review assessing stroke in periodontitis, gingivitis and tooth loss.

Study	Year	Country *	Number of Studies	Funding	Conflict of Interest	Quality Assessment
Janket et al. [20]	2003	United States of America	2	NA	NA	Moderate
Khader et al. [24]	2004	Jordan	6	NA	NA	Moderate
Mustapha et al. [16]	2007	United States of America	4	Yes	No	High
Sfyroeras et al. [25]	2012	Greece	13	No	No	Moderate
Lafon et al. [26]	2014	France	9	No	No	High
Leira et al. [27]	2017	Spain	8	Yes	No	High
Cheng et al. [28]	2018	China	8	No	No	High
Fagundes et al. [15]	2019	Brazil	11	NA	No	High
Larvin et al. [21]	2021	United Kingdom	19	Yes	No	High
Leng et al. [22]	2023	China	8	NA	No	High
Dewan et al. [23]	2023	India	19	No	No	Moderate

*: country of correspondence.

**Table 2:** Summary of the aims and findings of published systematic reviews and meta-analyses assessing stroke in periodontitis, gingivitis and tooth loss.

Study	Main Aim	Conclusion
Janket et al. [20]	To analyze the studies that provide a quantitative summary of the relationship between stroke and PD and explore possible causes for conflicting results.	Periodontal disease is associated with an increased risk of developing stroke in the general population, with the possibility of underestimation.
Khader et al. [24]	To examine the relationship between periodontal diseases and CVD in observational studies.	Periodontal infections (periodontitis and gingivitis) increase the risk of CVD.
Mustapha et al. [16]	To review the association between PD with elevated systemic bacterial exposure and CVD.	Periodontal disease has no consistent relationship with stroke, despite a possible relation to early carotid atherosclerosis.
Sfyroeras et al. [25]	To examine the relationship between PD and CVD in observational studies.	Periodontitis is associated with an increased risk of stroke. A combination of early prevention and treatment at a younger age and prevention of atherosclerotic risk reduces this risk.
Lafon et al. [26]	To determine the association between PD and stroke incidence.	Periodontitis and tooth loss are associated with the occurrence of stroke.
Leira et al. [27]	To investigate the possibility of an association between periodontitis and cerebral ischemia and its strength.	Antecedents of periodontitis may be a moderate to strong risk factor for ischemic stroke.
Cheng et al. [28]	To assess the relationship between tooth loss and risk of stroke.	Tooth loss is associated with deleterious stroke risk.
Fagundes et al. [15]	To investigate the association between Periodontitis and stroke.	Increased risk of stroke in patients with periodontitis, especially in ischemic events, and an association between the two diseases
Larvin et al. [21]	To examine the risk of incidents of stroke in people with PD in randomized controlled trials and longitudinal cohort studies.	Increased risk of stroke in PD populations by 24%.
Leng et al. [22]	To establish a link between stroke and periodontal disease and whether there is a sex difference.	An increased risk of stroke is associated with periodontal disease independent of sex.
Dewan et al. [23]	To review the association of periodontitis and gingivitis with various types of strokes.	Significant association exists between stroke and periodontal disease in case-control, cohort and cross-sectional studies.

**Table 3:** Tabular representation of the quantitative outcomes of the systematic reviews regarding the association between Periodontitis, gingivitis and tooth loss with stroke risk.

Oral Status	Systematic Review	Findings	Heterogeneity
Periodontitis	Janket et al. [20]	Significant correlation with stroke (RR = 2.85, 95% CI = 1.78, 4.56, *p* = 0.000).	I^2^ = NA
	Khader et al. [24]	Significant correlation with cerebrovascular diseases (RR = 1.13, 95% CI = 1.01, 1.27, *p* = 0.032)	I^2^ = NA
	Mustapha et al. [16]	No significant correlation with stroke (OR = 0.77, 95% CI = 0.37, 1.61, *p* = 0.49)	I^2^ = 79%
	Sfyroeras et al. [25]	Significant correlation with stroke in prospective studies (RR = 1.47, 95% CI = 1.13–1.92, *p* = 0.0036)	I^2^ = NA
		Significant correlation with stroke in retrospective studies (OR = 2.63, 95% CI = 1.59–4.34, *p*= 0.0002)	I^2^ = NA
	Leira et al. [27]	Significant correlation with ischemic stroke (RR = 2.88, 95% CI = 1.53–5.41, *p* = 0.000)	I^2^ = NA
	Fagundes et al. [15]	Significant correlation with CVA in Case-Control studies (95% CI = 1.39, 3.84, *p* = 0.001)	I^2^ = 77%
		Significant correlation with ischemic stroke in Case-Control studies (95% CI = 2.00, 3.71, *p* < 0.00001)	I^2^ = 4%
		Significant correlation with ischemic stroke cohort studies (95% CI = 2.00, 3.71, *p* < 0.00001)	I^2^ = 0%
	Larvin et al. [27]	Significant correlation with stroke (95% CI = 1.12, 1.38, *p* = 0.00)	I^2^ = 98.1%
	Leng et al. [22]	Significant correlation in males regarding stroke (OR = 1.29, 95% CI: 1.09–1.53). No significant sex difference regarding stroke (*p* > 0.05)	I^2^ = 79%
		Significant correlation in females regarding stroke (OR = 1.10, 95% CI: 1.09–1.11). No significant sex difference regarding stroke (*p* > 0.05).	I^2^ = 0%
	Lafon et al. [26]	Significant association between periodontitis and stroke (RR = 1.63, 95% CI = 1.25–2.00, *p* < 0.005)	I^2^ = NA%
	Dewan et al. [23]	Significant association between periodontitis and stroke (RR = 1.32, 95% CI = 1.04–1.60, *p* = 0.142)	I^2^ = 30.3%
Tooth loss	Khader et al. [24]	Significant correlation with cerebrovascular diseases (RR = 1.37, 95% CI = 1.10, 1.73, *p* = 0.006)	I^2^ = NA%
	Cheng et al. [28]	Significant association with stroke (MD = 0.15, 95% CI = 1.11, 1.25, *p* < 0.001).	I^2^ = 46.7%
	Lafon et al. [26]	Significant association with stroke (RR = 1.39, 95% CI = 1.13, 1.65, *p* < 0.005)	I^2^ = NA%
Gingivitis	Lafon et al. [26]	No significant association with stroke (RR = 1.10, 95% CI = 0.77, 1.65, *p* > 0.05).	I^2^ = NA%
	Dewan et al. [23]	No significant association between gingivitis and stroke (RR = 1.17, 95% CI: 0.42–1.92, *p* = 0.100)	I^2^ = 45.9%

## Data Availability

No new data were created or analyzed in this study. Data sharing is not applicable to this article.
